# Frequency effects in Spanish phonological speech errors: Weak sources in the context of weak syllables and words

**DOI:** 10.1017/s0142716423000231

**Published:** 2023-05-04

**Authors:** Julio Santiago, Elvira Pérez, Alfonso Palma, Joseph Paul Stemberger

**Affiliations:** 1 Universidad de Granada, Granada, Spain; 2 University of Liverpool, Liverpool, UK; 3 University of British Columbia, Vancouver, Canada

**Keywords:** speech errors, frequency effect, phonological encoding, Spanish, language production

## Abstract

The present study examines the effects of the frequency of phoneme, syllable, and word units in the Granada corpus of Spanish phonological speech errors. We computed several measures of phoneme and syllable frequency and selected the most sensitive ones, along with word (lexeme) frequency to compare the frequencies of source, target, and error units at the phoneme, syllable, and word levels. Results showed that phoneme targets have equivalent frequency to matched controls, whereas source phonemes are lower in frequency than chance (the Weak Source effect) and target phonemes (the David effect). Target, source, and error syllables and words also were of lower frequency than chance, and error words (when they occur) were lowest in frequency. Contrary to most current theories, which focus on faulty processing of the target units, present results suggest that faulty processing of the source units (phonemes, syllables, and words) is an important factor contributing to phonological speech errors. Low-frequency words and syllables have more difficulty ensuring that their phonemes, especially those of low frequency, are output only in their correct locations.

Human language is highly sensitive to the frequency of elements, such that high-frequency elements are processed more quickly and with greater accuracy than low-frequency elements (all other things being equal). This was originally demonstrated with single lexical items with no inflectional morphology (for a recent review, see Brysbaert et al., 2018) but has been extended to many other types of units (see below). In this paper, we will investigate the effects of frequency on different elements that participate in phonological errors occurring in natural speech production, and attempt to specify more precisely how different aspects of a word’s pronunciation are affected by frequency.

Current processing models are based on activation, with high-frequency words having greater activation levels than low-frequency words (e.g., Dell, 1986; Vitevitch & Luce, 2015). High-frequency words are faster because they more quickly reach the threshold level of activation needed for lexical access. Low-frequency words are more subject to error because, on some trials, they might be too slow to reach the activation threshold for access or, on other trials, they might be out-competed by a (faster) high-activation lexical item. Because an activated lexical item spreads activation to all its parts, high-frequency words also access their constituent phonemes more quickly and with less error (e.g. Dell, 1990; Stemberger & Macwhinney, 1986).

Arguments have arisen whether items that are fully predictable might be stored and hence might show similar frequency effects, such as high-frequency word strings. Umeda and Kahn (1982) observed that in the Francis and Kučera (1982) word frequency corpus, there are only 11 words that are more frequent than the word string *of the* and noted that it would be reasonable for such a high-frequency word string to be stored as a unit. Frequency effects (e.g., being faster on high frequency than on low frequency) have since been shown for word strings (e.g., Tremblay & Baayen, 2010). Such effects have been interpreted as showing that regularly inflected forms and word strings are stored, and some theories (e.g., Usage-Based Grammar; Bybee, 2006) presuppose that whole sentences are stored.

The phonological parallel to such complex items is syllables: phonemes such as /p/, /n/, /ei/, and /æ/ make up syllables such as /pei/, /nei/, /pæ/, and /næ/, and one can ask whether syllable units are stored and show frequency effects. There is ample evidence that syllables exist. For example, phonological speech errors show a strong parallel syllable position constraint: most contextual errors involving single phonemes and clusters respect syllable position (e.g., Nooteboom, 1969; Mackay, 1970), with onsets mostly interacting with onsets, nuclei with nuclei, and codas with codas. However, contextual errors in which whole syllables are mis-ordered are quite rare, leading to the point of view that syllables are structural frames which guide phoneme and segment insertion for production (e.g., Shattuck-Hufnagel, 1979), but that they are not themselves units, a viewpoint that predicts that syllables should not show frequency effects. Numerous studies have looked for effects of syllable frequency and for priming effects due to the use of syllable units, with some studies showing such effects (e.g., Chen et al, 2003; Laganaro & Alario, 2006; Levelt & Wheeldon, 1994; Álvarez, Carreiras & Perea, 2004; Carreiras & Perea, 2004; etc.), and others not (e.g., Nickels & Howard, 2004; Schiller, 1998; etc.). We will contribute to this literature by looking for effects of syllable frequency on phonological speech errors, asking whether such effects are parallel to the effects of word frequency and phoneme frequency.

Speech errors have characteristics that allow us to make more precise what sorts of properties can be in error. Most lexical errors are paradigmatic or noncontextual in nature: they involve just the target item and whether it is accurately produced. Most phonological errors, in contrast, are syntagmatic or contextual in nature: they involve interference between two items in an utterance, where at least one item is produced in the wrong location, and most often where that item is produced twice, in both the correct and error locations (e.g., Dell & Reich, 1981; Nooteboom, 1969; Stemberger, 1991a). All theories predict that, if there is any effect of frequency on noncontextual paradigmatic errors, it will be a lower error rate on high-frequency targets than on low-frequency targets. For contextual syntagmatic errors, spreading activation theories predict that the system is biased to output strong units (units that reach the threshold for selection quickly) at the expense of weak units, that is, strong units are expected to out-compete weak units. This bias to output strong units should hold at all levels: phonological features, phonemes, syllables (at least if they are stored), and words. This study allows us to test whether the output bias toward strong units is as broad as usually supposed, or whether it holds just on some aspects of errors.

An example will serve to clarify the vocabulary that we will use henceforth to refer to the units involved in a single-phoneme speech error:

(1) Siempre está ab***u***erta la p**u**erta.

– Siempre está ab***i***erta la p**u**erta.

“The door is always open.”

In this example, at the phoneme level the *error* unit is the /u/ in /abuerta/, the *source* unit is the /u/ in /puerta/, and the *target* unit is the intended /i/ in /abierta/. At the syllable level, the error unit is the syllable /buer/, the source unit is /puer/, and the target unit is /bier/. At the word level, the error unit is /abuerta/, the source unit is /puerta/, and the target unit is /abierta/.

Applied to single-phoneme contextual errors, spreading activation theories predict that source phonemes should be of higher frequency than target phonemes, the better to out-compete them. Henceforth, we will call high(er) frequency units *strong* units and low(er) frequency units *weak* units. Frequency effects should manifest in both the competition between the relevant units in a speech error (the source and the target, resulting in the error) and in the contrast between the frequency of these units and the frequency they should have by chance. The stronger source phoneme should win the competition with the target phoneme and surface as the error phoneme, and thus both the source and error phonemes should have higher frequency than the target. In other words, strong source phonemes do not affect randomly chosen target phonemes but preferentially those which are weaker. When compared with chance, source and error phonemes could be at chance frequency or above and the target phoneme should be a weak unit: of lower frequency than chance.

In spreading activation networks, upper levels (such as words) spread activation downwards to their constituent units, increasing the strength of those units. If there is a syllable layer between words and phonemes, source phonemes should originate in syllables which are of equal or higher frequency than the syllables that contain the target phoneme. When a single phoneme moves, the error syllable is different from the target and the source, but the same logic applies: the syllable actually produced (in error) should be of equal or higher frequency than the target syllable. When compared with chance, the target syllable is expected to be a weak unit (lower frequency than chance) whereas the source and error syllables should be at chance or above. Finally, source phonemes should also come from word units that are as strong or stronger than those containing the target phonemes.^1^

Finally, there is the issue of whether there is an effect of frequency that manifests on the error word itself. It has long been noted that phonological errors can by chance resemble real words (e.g., in the phrase *pick a fig*, if the /p/ of *pick* were to perseverate and replace the /f/ of *fig*, the real-word outcome *pig* would result: *pick a **p**ig*). Motley et al. (1983) argued that there is a lexical bias on phonological speech errors, such that real-word outcomes are more frequent than expected by chance. Dell (1986) and Dell and Reich (1981) explained the lexical bias as arising from feedback from the erroneous phoneme back to the word level, secondarily activating the error word, which sends back activation to reinforce the error; in this way, there is a bias to produce outputs that are well-formed at many levels. While not addressed in the literature, it would seem reasonable that high-frequency words would contribute more activation than low-frequency words, so that phonological errors that lead to high-frequency real-word outcomes should be more common than errors that lead to low-frequency real-word outcomes. It should be noted, however, that there is no lexical bias in the Granada corpus that we are using here (Pérez et al, 2007) or in the Oviedo corpus of Spanish speech errors (del Viso, 1992; del Viso et al., 1991). That is, there is no evidence in naturalistic corpora of Spanish speech errors that the proportion of word outcomes is greater than expected by chance, which has implications for our discussion below.

We will use the term *Strong Unit effect* to refer to cases in which a given unit is of greater frequency than expected by chance (e.g., a Strong Source effect will mean that sources are of greater frequency than chance) and its converse *Weak Unit effect* to refer to cases in which a given unit is of lower frequency than chance (e.g., a Weak Target effect). When error, target, and source units are all of different frequency than their controls, there may be a combination of Strong Unit and Weak Unit effects. We will also give a proper name to the direct competition effect: if, as expected, sources are of greater frequency than targets, we will call this a *Bully effect*.

We focus on word, syllable, and phoneme frequency in this paper but will not directly address feature frequency, whether higher-frequency features such as [Coronal,+anterior] in /t, d, s, n/ behave differently than lower-frequency features such as [Labial] in /p, b, f, m/ or [Dorsal] in /k, g, x/. Most phonological speech errors involve whole phonemes, though features importantly determine similarity between phonemes (e.g., Dell & Reich, 1981; Nooteboom, 1969; Stemberger, 1991b, 2009). Shattuck-Hufnagel and Klatt (1979) argued that there were no effects of phoneme and feature frequency, with some oddities such as a bias for high-frequency anterior-coronal /s/ to be replaced by low-frequency nonanterior-coronal /ʃ/. Using an experimental task with nonwords, Levitt and Healy (1985) reported not only no “palatal bias” but also no phoneme-frequency or feature-frequency effects. Stemberger (1991a) reported a more complex picture for these syntagmatic errors: when the two competing features were of the same type (both place, both manner, or both voicing), the element of higher frequency was favored for medium-frequency and lower-frequency features (e.g., [Labial] vs. [Dorsal] place), but the highest-frequency feature ([Coronal] place, the “default” place feature) was disfavored (which Stemberger called the *nondefault bias*). Stemberger argued that this is a property that emerges from an interaction of frequency with error-driven learning, involving issues too complex to address here. Because a simple frequency effect is not predicted, we will take the nondefault bias into account as a covariate in our analyses of phoneme frequency (based on which feature is favored or disfavored), to see whether it has a significant effect in our data and statistically control for it.

To summarize, the expectation of spreading activation theories is that target, source, and error units of speech errors will differ in frequency, both when compared to chance and between themselves. (1) Errors are expected to occur more on weak (low-frequency) target units, because they are processed more poorly: target units should statistically be of lower than chance frequency (a Weak Target effect). (2) If there is an effect of frequency in what the speaker actually produces (the error), there should be an advantage for strong units: the contributions of the lexicon and the phonological system should favor high-frequency output units, so error units should statistically be of higher than average frequency (a Strong Error effect). (3) The intruding source units should be of higher than average frequency (a Strong Source effect), or at least of higher frequency than target units (a Bully effect), to more effectively intrude on a more poorly processed target unit. We will show that many of these expectations do not hold true in our data.

The present study addresses frequency effects in a large naturalistic database: a corpus of over 8000 spontaneous speech errors produced by neurologically healthy Spanish adults, which was collected at the University of Granada. Looking at naturalistic speech errors improves the generalizability of findings to speech in everyday settings. Using this corpus, we explore frequency effects at the phoneme, syllable, and word levels on single-phoneme speech errors, including error, target and source units, and suitable chance conditions, using a variety of different frequency indices. We first determine which is the most representative frequency measure and then search for effects of word, syllable, and phoneme frequency while controlling for other factors.

## Methods

### The speech error corpus

The source of our data is a corpus of 8031 naturalistic Spanish speech errors collected over several years by over 737 students at the University of Granada, Spain, under the supervision of one of the authors (Palma). A detailed description of the training and collection methods, and control procedures can be found in Pérez et al. (2007). The Granada corpus is a multiple-collector effort, drawing on a large number of theoretically naive observers. It might be argued that this method of error collection is more susceptible to perceptual biases than the more common single-collector procedure, in which a small number of highly trained theoretically informed observers provide most of the errors in the corpus. Pérez et al. (2007) carried out a cross-validation of the present corpus with the single-collector Oviedo corpus of Spanish speech errors (del Viso, 1992; del Viso et al., 1991) and found none of the effects predicted if multiple-collector corpora magnify the effects of perceptual bias. In any case, the type of effects that we will be looking for in the present research, especially those on the source units, is unlikely to arise as a result of perceptual bias. The present investigation uses all phonological errors collected up to the year 2001 (*N* = 1477).

Ninety-three of the total 1477 phonological errors were discarded because they changed the total number of syllables of the target word, a requirement imposed by the way that our syllable frequency measures are calculated (see below). Additionally, 406 errors were also discarded because they involved more than one phoneme, therefore precluding the calculation of phoneme frequency. Another important factor in the analyses to follow is whether the source unit can be unambiguously located. For example, exchanges always have unambiguous sources, as in the following example:

(2) Que me des el perió***k***i***d***o

– Que me des el perió***d***i***c***o

“Give me the newspaper”

Anticipatory substitutions, perseveratory substitutions, and shifts without loss of the source unit may have an ambiguous source if there are two or more candidates in the surrounding context. Example (3) is an unambiguous anticipatory substitution, whereas (4) is an ambiguous error, with a possible source at the beginning of the same word and two other possible sources three words later:

(3) Me ba***ch***o en he**ch**os.

– Me ba***s***o en hechos.

“I rely on facts.”

(4) ¿Quién va a ser el **p**ilo***p***o, el tío **P**e**p**e o tú?

— ¿Quién va a ser el **p**ilo***t***o, el tío **P**e**p**e o tú?

“Who is going to be the pilot, uncle Pepe or you?”

Thus, from the remaining 978 errors, we excluded additional 414 errors with an ambiguous source. The remaining 564 errors comprised 218 anticipations, 128 perseverations, and 218 exchanges, all of them involving a single phoneme with an unambiguous source.

Another important factor to be controlled for is the number of phonemes in the syllable, because of the strong negative correlation between the length and the frequency of linguistic units (Zipf, 1949). Some errors do not change the length of the target syllable (anticipatory, perseveratory, or noncontextual substitutions and exchanges), whereas others do (e.g., shifts). As we are including only single-phoneme errors in the analyses, target and error syllables (and their controls) are matched in length, but target and source syllables may not have equal length. We controlled for this factor in the present analyses by selecting only those cases involving target and source syllables of equal length, what left a total *N* = 361 speech errors (139 anticipations, 75 perseverations, and 147 exchanges). Finally, three exchanges had to be removed because of experimenter or computer error. This left 144 exchanges in the dataset.

The same approach was used with respect to other factors that may be relevant to particular comparisons at the word or phoneme levels. For example, not all errors can be used to look at the lexical frequency of the error word (as phonological errors often result in nonwords). To deal with these cases, suitable subsets of errors were selected depending on the hypothesis being tested. Finally, some variables were controlled for by means of statistical procedures, introducing them as additional predictors into multiple regression analyses. This approach was used to statistically account for the effect of the frequency of units at the other levels of analysis (word, syllable, and phoneme) and for the effect of phonological underspecification at the phoneme level.

Several comments are in order. First, note that both halves of exchange errors were included in the analyses independently (as by, e.g., Stemberger, 2009), thus doubling the number of data points contributed by exchanges (288 cases, making the total *N* = 502). This may seem a questionable decision given that one of our goals is comparing the frequencies of the target and source units. In an exchange, the exchanging units take both the target and source roles, so introducing them twice in the analysis alternating their roles seems unwise. However, it must be emphasized that this decision can only work against the finding of significant differences in the frequency of target and source units. By definition, in exchanges the two units will balance each other out at all levels (phoneme, syllable, and word), just adding noise to the data. We decided to keep exchanges in the analyses in order to avoid losing statistical power in other relevant comparisons (e.g., between the target unit and its control), which are not affected by such noise (additionally, the supplementary materials include analyses using only the anticipatory side of exchanges that parallel in all respects the results reported hereby). A similar problem occurs when the frequencies of target and source words are compared when the phonological error occurs within a word. In this case, the target and the source words are the same, so these errors contribute identical numbers to each side of the comparison (note that the problem only occurs at the word level, as within-syllable errors are extremely rare and unattested in our corpus). Again, we opted for leaving these speech errors in the designs, as they only contribute to the variability of the random factor, which is duly controlled for by the linear mixed models.

### The Granada Lexical Database

Both the frequency measures and the procedures for chance estimation rely on the use of a lexicographic tool named the Granada Lexical Database (GRLDB), developed from the Alameda and Cuetos (1995) lexical database and frequency count. This is a highly curated word corpus based on written nononline sources (books, newspapers). Their lexical corpus contains 81,313 word types (orthographic lexemes) from a sample of two million word tokens. The index of lexical frequency is therefore lexeme (not lemma) frequency. Words in the database were phonologically transcribed and syllabified using an automated procedure described in Santiago et al. (1996) for their LEX II database. This procedure assigns a single character to each phoneme relying on the shallow orthography of Spanish and a small set of orthographic rules. Because of this, it does not distinguish allophones (e.g., word-initial vs. medial oclusives). This is probably the correct level of analysis for phonological speech errors, given the abundant evidence for allophonic adaptation of phonemes to their new context after they get involved in an error (e.g., Fromkin, 1971). The following information was then added to each lexical entry: number of syllables, primary stress location, CV structure, number of lexical neighbors, and average frequency of lexical neighbors. The phonological syllables of each word were placed into a positional frame of 10 positions, from “1” to “last.” For example, a bisyllabic word has entries in the first and last syllabic fields. Monosyllables fall into the last position. Each syllable also has a field indicating its stress level (0 for unstressed, 1 for stressed) and another with its CV structure.

### Measures of frequency and phonological default/nondefault status

Only one index of lexical frequency was used, the orthographic lexeme count provided by Alameda and Cuetos (1995).

Twelve different indexes were computed for syllable frequency (see an example in Table 1), resulting from the combination of the following dimensions:

1. *Type versus token frequency:* The type frequency of a syllable counts how many different words contain that syllable. Token frequency results from adding up the lexical frequencies of those words.
2. *Positional frequency in an absolute word frame:* This is a common frame for all words, distinguishing positions first to ninth and last.
3. *Positional frequency in a relative frame:* This measure uses different positional frames for each word length in syllables.
4. *Nonstress versus stress-sensitive:* Stress-sensitive measures treat the different stress values of the same syllable as different syllables.

There was a type and a token frequency count for each positional measure and for the stress-sensitive measure, as well as absolute type and token counts, what rendered eight frequency measures (bottom eight rows in Table 1). Moreover, there were stress-sensitive type and token counts for each positional measure, rendering four more measures (top four rows in Table 1).

Four frequency indexes of phoneme frequency were computed (see Table 2), resulting from crossing two dimensions:

1. *Type versus token frequency:* Phoneme type frequency counts the number of occurrences of that phoneme in different syllables. Token frequency adds up the absolute token frequency of those syllables.
2. *Positional frequency in an absolute syllable frame:* This is a common frame for all syllables with two onset positions, three vowel nucleus positions, and two coda positions. These are all the syllabic positions allowed by Spanish phonology.

In order to be able to control for the presence of a nondefault bias in the set of single phoneme errors, we used feature matrices listing only the (unpredictable) nondefault features for Spanish, shown in Table 3 (consonants) and Table 4 (vowels), based on tables for English in Bernhardt and Stemberger (1998). In Spanish, the default features are those of the consonant /t/ and the vowel /e/. Note that “+” and are used for binary features, and “√” denotes the presence of a privative (single-valued) feature or organizing node. Default and predictable features are left out of the feature matrix, because it has been argued that they have more diffuse representations in language production and are only weakly represented in individual lexical entries. An index of phonological underspecification was devised for each single-phoneme error by counting the number of underspecified features (i.e., the higher the index, the less specified are the features for the phoneme). Table 5 shows the Spanish phonemes together with their absolute token frequency and the number of times in which they participated as source and as targets in the sample of speech errors used for the present analyses.

### Estimation of chance

Our approach to chance estimation differs from prior methods (see Stemberger, 1991b, for a review). Working from the GRLDB, we were able to randomly select a control unit from all available units of that kind in GRLDB for each relevant (error, target, or source) word, syllable, and phoneme, such that the control unit was matched in as many relevant parameters as possible. Word controls were matched with the relevant word in number of syllables and stress position. Syllable controls were matched with the relevant syllable in CV structure, stress level, and position in a frame relative to the word’s number of syllables. Phoneme controls were matched with the relevant phoneme in syllable position in an absolute frame with two onset positions, three vowel nucleus positions, and two coda positions. Within the set of matched controls for each relevant unit, random extraction took into account a priori probabilities based on the frequency of the candidates in the most specific frequency measures. Table 6 shows a complete record from our data file, corresponding to the vowel anticipation in (1).

In this method of chance estimation, the control unit is randomly selected from the set of potential tokens (not types) that match the relevant unit in the selected parameter values. This is necessary in order to avoid a bias toward low-frequency types. At all levels, linguistic types follow a power law distribution, such that a few types have very high frequencies (there are many tokens of those types), and frequency drops sharply asymptotically approaching zero, such that a great number of types occur very rarely (i.e., they are represented by very few tokens). If control extraction is done at the level of types, the selected unit would be most often a low-frequency type. In the present study, we avoided this problem by carrying out extraction at the level of tokens.

When this selection process is repeated over a large sample of units, it behaves like a Monte Carlo simulation of the frequency distribution of those units in the lexicon. In this way, the frequency distributions of relevant and control units can be compared. This procedure resembles that used by Stemberger and MacWhinney (1986), who identified the point that divided the lexical frequency distribution into two halves, leaving 50% of word tokens at each side, and then tabulated how many target words belonged to the higher or lower frequency sets. An advantage of the present procedure is that it does not use the whole frequency distribution but just the distribution of those units which are matched to the relevant unit in some important parameters (e.g., at the word level: number of syllables and stress pattern).

All frequency indexes were computed for each relevant unit and its matched control. We first reduced the multiple indexes of syllable and phoneme frequency to the most representative index. We then carried out linear mixed models on that index with the goal to compare unit frequency across the three relevant types of units (error, target, and source) at the three levels (word, syllable, and phoneme), as well as to compare each relevant unit to a chance estimation.

It might be argued that the present chance estimation procedure is not well suited for some of these contrasts. For example, the control syllable selected for the error syllable /buer/ in Table 6 is /gien/. However, the syllable /gien/ is unlikely to be active in this particular error and so, it should not be considered a potential replacement for /buer/. This argument, though, fails to consider that the chance estimation procedure works over the whole sample of errors. To put it differently, the comparison is between the observed set of error syllables and a set of control syllables of closely matched syllable structure, stress level, and word position. The selection process of control units secures that, as a group, the control units are matched in important characteristics with units involved in speech errors (either as a source, target, or error). As the set of control units is a randomly extracted sample from the set of units in the language that share with the speech error unit important characteristics, we can use the control set to estimate what is the average frequency that should be expected by chance. The best way to conceive of this strategy of estimation of chance is as a comparison between the whole set of units involved in a speech error (source, target, or error) and its control set for a given kind of unit (phoneme, syllable, word).

Although we agree that it would be better to be even more precise and impose further constraints on the selection of the control set (including, e.g., phonological similarity), the current procedure already outperforms available alternative methods. Moreover, the criticism does not apply to all contrasts. For example, comparisons between the source unit and its control and between the target unit and its control are free from this criticism because the controls are sampled, in principle, from the same underlying distribution as the relevant units. Comparisons between error, target, and source units are also free from this criticism because they do not involve control units. Most of the findings reported hereby concern these safe contrasts. For the rest (contrasts involving the error unit), they should be taken as the best available approximation, until even more precise methods of chance calculation are devised.

## Results

Our main goal was to explore frequency effects across error, target, and source units and their control conditions at the levels of the phoneme, the syllable, and the word, while factoring out a number of potential confounds. Our first step in that direction was to find out the most representative frequency measure of syllable and phoneme frequency out of the 12 syllable and 4 phoneme measures. We then turned to linear mixed models to examine effects of frequency at each level (phoneme, syllable, and word) while statistically controlling for correlations with variables at other levels. All analyses reported in the present paper were carried out using R (R Core Team, 2018). The raw data, the analysis script for all the reported and additional analyses, tables and figures, and supplementary information are publicly available at https://osf.io/u3cwr.

### Principal component analysis of frequency measures at syllable and phoneme levels

Details on how the principal component analysis allowed us to single out one measure of syllable and one measure of phoneme frequency are described in the Supplementary Analyses document. For syllable frequency, the analysis suggested the use of the token syllable frequency in a 10-syllable word frame (i.e., disregarding the stress value of the syllable and the total number of syllables of the word). For phoneme frequency, it suggested the use of token phoneme frequency in a syllable-position frame. Thus, the results reported below are based on these two measures. However, it was also clear from the analysis that the different ways to calculate syllable and phoneme frequency are roughly comparable and equally valid. The supplementary analysis script contains commands to carry out the same analyses reported herein on several alternative measures: the pattern of results was the same in all of them.

### Frequency effects at phoneme, syllable, and word levels

#### Designs and analysis

To analyze frequency effects in single-phoneme speech errors, we used three designs: the Overall Design, and the Partial Designs A and B. The Overall Design included two fully crossed fixed factors: Unit Type (error, target, and source) and Key-Control (key unit vs. control unit). Three independent analyses of the Overall Design were carried out at each Level (phoneme, syllable, and word). The unit of analysis (the random factor) was speech error. All fixed factors varied within the unit of analysis (repeated measures). Frequency was the dependent measure: at the word level it was lexeme frequency, at the syllable level it was positional token frequency in an absolute word frame, and at the phoneme level it was positional token frequency in an absolute syllable frame.

The dependent variables were log_10_ transformed to adjust their distribution to linear regression requirements. This introduced a problem with those target and source words that were unattested in GRLDB (20% of target words and 17.9% of source words), because their frequencies were set to zero in the datafile, and thus, their logarithm could not be computed. Error words pose a more difficult problem, as they often result in true neologisms. We went through the whole set of error words and marked those that were real words but unattested in GRLDB (7.7% of all error words). In some cases, syllable frequencies generated the same problem. The Spanish syllabary being relatively limited, there were only two cases of errors creating true syllable neologisms (one is /buer/ in the example above), but there were some error syllables that were unattested in GRLDB at that particular word position and/or stress level, generating a frequency of zero in some of the more specific syllable frequency measures (the maximum was 3.1% zero frequencies in the most specific syllable frequency; the supplementary script contains commands to compute the incidence of zero frequencies in all frequency measures). To deal with the problem of zero frequencies, we used a Laplace transformation (as recommended by Brysbaert & Diependaele, 2013), adding 1 to the frequency count of all types in each measure of both word and syllable frequency. There were no zero frequencies in any of the phoneme-frequency measures, so they were not corrected.

The Overall Design included only those cases that result in real words (as nonword outcomes do not have a lexical frequency). This leaves us with only 130 cases (38 anticipations, 17 perseverations, 36 anticipatory sides of exchanges, and 39 perseveratory sides of exchanges) and does not let us test the important set of cases resulting in nonwords.

In order to increase statistical power and include the many errors resulting in nonwords in the design, we carried out two analyses of partial designs. Since the problem is caused by the cell containing the frequency of error word units, the partial designs circumvented this cell. Partial Design A contained all three Levels (phoneme, syllable, and word) and the Key-Control contrast, but only two Unit Types (target and source, eliminating error units). Partial Design B focused on only two Levels (phoneme and syllable, eliminating word units), plus the Key-Control contrast and all three Unit Types (error, target, and source). The resulting *N* in the two partial designs is 502 cases (139 anticipations, 75 perseverations, 144 anticipatory sides of exchanges, and 144 perseveratory sides of exchanges).

We report results of linear mixed model analyses on the frequency measure at each Level (phoneme, syllable, and word). The random-factor structure of the model included random intercepts for each speech error (remember that the two sides of exchanges were entered into the analysis as independent speech errors). The fixed-factor structure included the factors Unit Type (error, target, and source) and Key-Control (key vs. control unit) as categorical predictors and the frequency measures from the other levels as continuous predictors. The analysis at the phoneme level also included the degree of phonetic feature underspecification in the model. Categorical predictors were dummy-coded and centered, and sum contrasts were used to allow for ANOVA-like interpretation of results. Analyses without the covariate of phoneme underspecification, without any covariates, and using only the anticipatory side of exchanges are reported in the supplementary materials. Linear mixed models were computed using lme4 (Bates et al, 2015). Their results were converted to ANOVA format for easier reporting by means of the function anova() from the LmerTest package (Kuznetsova et al., 2017) using Satterthwaite’s method. Effect sizes (partial eta squared) were computed from the F values and degrees of freedom using the effectsize package (Ben-Shachar et al., 2020). Post-hoc comparisons using Tukey correction were computed with the package emmeans (Lenths, 2019).

#### Overall Design: Results

**Figure 1.**
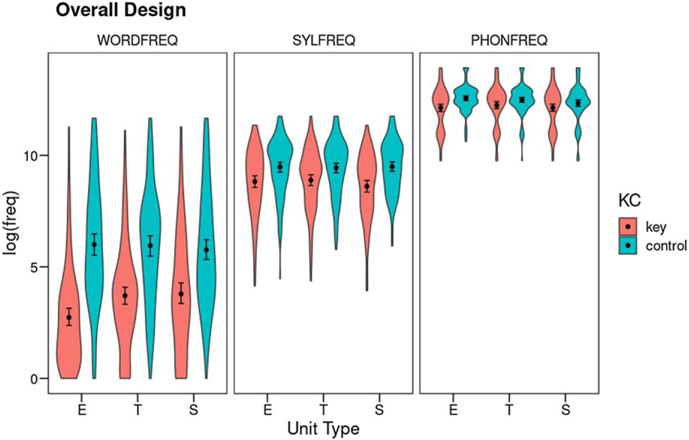
Overall Design: Mean log_10_ frequency for the factors Unit Type (E: error; T: target; S: source), Key-Control, and Level (WORDFREQ: word frequency; SYLFREQ: syllable frequency, PHONFREQ: phoneme frequency). Error bars show 95% confidence intervals using nonparametric bootstrap. The violins show the density of the distributions.

The Overall Design includes independent analyses at all three levels: phoneme, syllable, and word. At each level, the design includes three Unit Types (error, target, and source) and their contrast with matched controls (Key-Control). This design includes only cases that result in real words. Total *N* is 130 cases. Figure 1 shows cell means, 95% nonparametric confidence intervals, and the shapes of the distributions (see also Table S3 for cell means and SDs).

##### Phoneme frequency:

Phoneme frequency was analyzed as a function of the factors Unit Type (error, target, and source) and Key-Control (key units vs. control units), controlling for word and syllable frequency as well as the number of underspecified features in the involved phonemes (Table S4 in the supplementary materials reports the full output of the model). Regarding the covariates, word frequency did not account for a significant portion of variance (*F*(1,770.69) = 0.04, *p* = .84, *η*^2^_*p*_<.01) while both syllable frequency (*F*(1,770.58) = 67.13, *p* < .001, *η*^2^_*p*_ = .08) and phoneme underspecification (*F*(1,700.33) = 7.72, *p* = .006, *η*^2^_*p*_ = .01) did. The analysis of the factors of interest revealed a Weak Units effect: Key phonemes were of lower frequency than their matched controls (*F*(1,704.94) = 8.65, *p* = .003, *η*^2^_*p*_ = .01). There was no effect of Unit Type (*F*(2,643.73) = 2.06, *p* = .13, *η*^2^_*p*_ < .01), and no interaction between Key-Control and Unit Type (*F*(2,644.71) = 2.78, *p* = .06, *η*^2^_*p*_ < .01), although it approached significance.

##### Syllable frequency:

Syllable frequency was analyzed as a function of Unit Type (error, target, and source) and Key-Control (key units vs. control units), controlling for word and phoneme frequency (see Table S5 for the full output of the model). Both covariates had significant effects on syllable frequency (word frequency: *F*(1,771.95) = 17.85, *p* < .001, *η*^2^_*p*_ = .02; phoneme frequency: *F*(1,771.97) = 70.66, *p* < .001, *η*^2^_*p*_ = .08). Regarding the effects of the factors of interest, at the syllable level there was also a Weak Units effect: Key syllables were of lower frequency than their matched controls (*F*(1,705.49) = 16.58, *p* < .001, *η*^2^_*p*_ = .02). There was no effect of Unit Type (*F*(2,645.04) = 0.34, *p* = .71, *η*^2^_*p*_ < .01), but the interaction between Key-Control and Unit Type was significant (*F*(2,645.88) = 3.15, *p* = .04, *η*^2^_*p*_ < .01). Post-hoc Tukey-corrected comparisons were used to explore the interaction. Source syllables were of lower frequency than their controls (a Weak Source effect, *t*(1,660) = −4.64, *p* < .001), whereas neither target syllables nor error syllables were of lower frequency than their controls (target: *t*(1,665) = −1.93, *p* = .39; error: *t*(1,686) = −1.35, *p* = .76). There were no differences between the three key units (target, source, and error) nor between the three control units.

##### Word frequency:

Finally, word frequency was analyzed as a function of Unit Type (error, target, and source) and Key-Control (key units vs. control units), controlling for syllable and phoneme frequency (see Table S6 for the full output). Syllable frequency accounted for a significant portion of variability in word frequency (*F*(1,771.76) = 17.90, *p*<.001, *η*^2^_*p*_ = .02), but phoneme frequency did not (*F*(1,771.79) = 0.18, *p* = .68, *η*^2^_*p*_<.01). Word frequency showed a significant Weak Units effect, with key words being of lower frequency than matched controls (*F*(1,664.57) = 219.84, *p*<.001, *η*^2^_*p*_ = .25), paired with a significant main effect of Unit Type (*F*(2,643.41) = 4.12, *p* = .02, *η*^2^_*p*_ = .01) qualified by an interaction between Key-Control and Unit Type (*F*(2,643.79) = 7.62, *p*<.001, *η*^2^_*p*_ = .02). Post-hoc Tukey-corrected comparisons showed that all key units were of significantly lower frequency than their corresponding controls, while control words for error, target, and source words did not have different frequencies. The interaction arose because the frequency of the error word was lower than the frequency of the target word (*t*(1,644) = −3.75, *p* = .003) whereas the latter did not differ from the source word (*t*(1,644) = −0.62, *p* = .99).

Summing up, this analysis supported the existence of frequency effects at all three levels which cannot be accounted for either by cross-covariations between frequency measures or by the degree of phonetic feature underspecification of the involved phonemes. The analysis of the Overall Design showed that at phoneme, syllable, and word levels, units involved in speech errors were of lower frequency than expected by chance, what we have called a Weak Units effect. At the syllable level, this effect was stronger on the source syllable (a Weak Source effect) than on target and error syllables. At the word level, the word resulting from the phonological error was of lower frequency than the target word. However, several facts suggest that this analysis may be somewhat underpowered: firstly, the interaction at the phoneme level approached significance, and secondly, the overall Weak Units effect at the syllable level was only substantiated by post-hoc comparisons on the source unit. By including the speech errors that resulted in nonwords, the following two partial designs provided complementary analyses with greater statistical power, at the cost of losing the ability to evaluate any effects on error word frequency.

#### Partial Design A (without the error unit): Results

**Figure 2.**
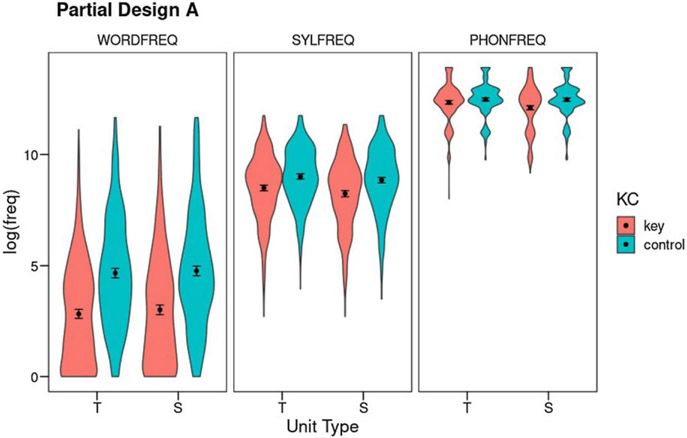
Partial Design A: Mean log_10_ frequency for the factors Unit Type (T: target; S: source), Key-Control, and Level (WORDFREQ: word frequency; SYLFREQ: syllable frequency, PHONFREQ: phoneme frequency). Error bars show 95% confidence intervals using nonparametric bootstrap. The violins show the density of the distributions.

Partial Design A contains only two Unit Types, target and source. At each Level (phoneme, syllable, and word), Unit Types are factorially crossed with the Key-Control contrast, with a total *N* of 502 cases. By including both errors resulting in words (*N* = 130) as well as nonwords (*N* = 372), it affords greater statistical power to assess the Weak Units effect as well as its potential interactions with target and source unit types, although the absence of error units in this design precludes the assessment of any potential effects on the error phoneme, syllable, or word. Figure 2 shows the results (see also Table S7 for means and SDs).

##### Phoneme frequency:

Phoneme frequency was analyzed as a function of Unit Type (target vs. source) and Key-Control, controlling for the degree of phoneme underspecification, and syllable and word frequencies (see Table S8 for the full output). All three covariates accounted for significant portions of variance (phoneme underspecification: *F*(1,1728.4) = 44.19, *p* < .001, *η*^2^_*p*_ = .02; syllable frequency: *F*(1,1991.2) = 142.04, *p* < .001, *η*^2^_*p*_ = .07; word frequency: *F*(1,1999.2) = 3.65, *p* = .06, *η*^2^_*p*_ < .01). Regarding the factors of interest, at the phoneme level there was an overall Weak Units effect, as key units were of lower frequency than their controls (*F*(1,1660.2) = 31.55, *p* < .001, *η*^2^_*p*_ = .02). There was also a Unit Type effect (*F*(1,1502.3) = 8.23, *p* = .004, *η*^2^_*p*_ < .01), qualified by a significant interaction between both factors (*F*(1,1496.5) = 13.25, *p* < .001, *η*^2^_*p*_ < .01). Post-hoc Tukey tests revealed a Weak Source effect: the source phoneme was of lower frequency than its matched control (*t*(1,1590) = −6.61, *p* < .001), but target phonemes were not lower in frequency than controls (*t*(1,1592) = −1.74, *p* = .31). Moreover, the source phoneme was of lower frequency than the target phoneme (*t*(1,1505) = 4.60, *p* < .001). By analogy with the biblical story of little David defeating giant Goliath, we will call this the David effect. Both control units were of comparable frequency (*t*(1,1502) = −0.54, *p* = .95).

##### Syllable frequency:

Syllable frequency was analyzed as a function of Unit Type (target vs. source) and Key-Control, controlling for phoneme and word frequencies (see Table S9 for full output). Both covariates were significant (phoneme frequency: *F*(1,2001.3) = 145.64, *p* < .001, *η*^2^_*p*_ = .07; word frequency: *F*(1,1984.3) = 65.12, *p* < .001, *η*^2^_*p*_ = .03). The analysis of the factors of interest revealed a Weak Units effect (*F*(1,1650.2) = 22.05, *p* < .001, *η*^2^_*p*_ = .01). The factor Unit Type was also significant (*F*(1,1497.2) = 10.74, *p* = .001, *η*^2^_*p*_ < .01): source syllables and their controls were of lower frequency than target syllables and their controls. However, the evidence for a David effect is unclear, because the two factors failed to interact significantly (*F*(1,1495.6) < 0.001, *p* = .99, *η*^2^_*p*_ < .01). Moreover, post-hoc comparisons only showed significant differences between each key unit and its control but not between the target and source key units (*t*(1,1512) = 2.30, *p* = .10). The target and source control units did not differ (*t*(1,1501) = 2.34, *p* = .09).

##### Word frequency:

Word frequency was analyzed as a function of Unit Type (target vs. source) and Key-Control, controlling for syllable and phoneme frequencies (see also Table S10). Both covariates had significant effects (phoneme frequency: *F*(1,2000.2) = 4.45, *p* = .03, *η*^2^_*p*_ < .01; syllable frequency: *F*(1,2000.9) = 61.17, *p* < .001, *η*^2^_*p*_ = .03). Word frequency showed a pattern of results that was similar to the syllable level. There was again a clear Weak Units effect (*F*(1,1533.6) = 349.25, *p* < .001, *η*^2^_*p*_ = .19) accompanied by a much smaller effect of the factor Unit Type (*F*(1,1502.1) = 4.58, *p* = .03, *η*^2^_*p*_ < .01), without a significant interaction between the two (*F*(1,1499.7) = 0.25, *p* = .62, *η*^2^_*p*_ < .01). Post-hoc tests again found differences between key and control units both for targets and sources, but not between targets and sources (key units: *t*(1,1511) = −1.86, *p* = .25; control units: *t*(1,1503) = −1.17, *p* = .65).

Partial Design A focused on target and source units and the key versus control contrast across the three linguistic levels (phoneme, syllable, and word). A Weak Units effect was observed at both the word and syllable levels, with both target and source units involved in speech errors being less frequent than expected by chance. However, at phoneme level we found that targets were at chance frequency, but they were being replaced by lower-frequency source phonemes, an effect that we called the David effect. In a somewhat less statistically clear way, this effect was also present at syllable and word levels.

#### Partial Design B (without the word level): Results

**Figure 3.**
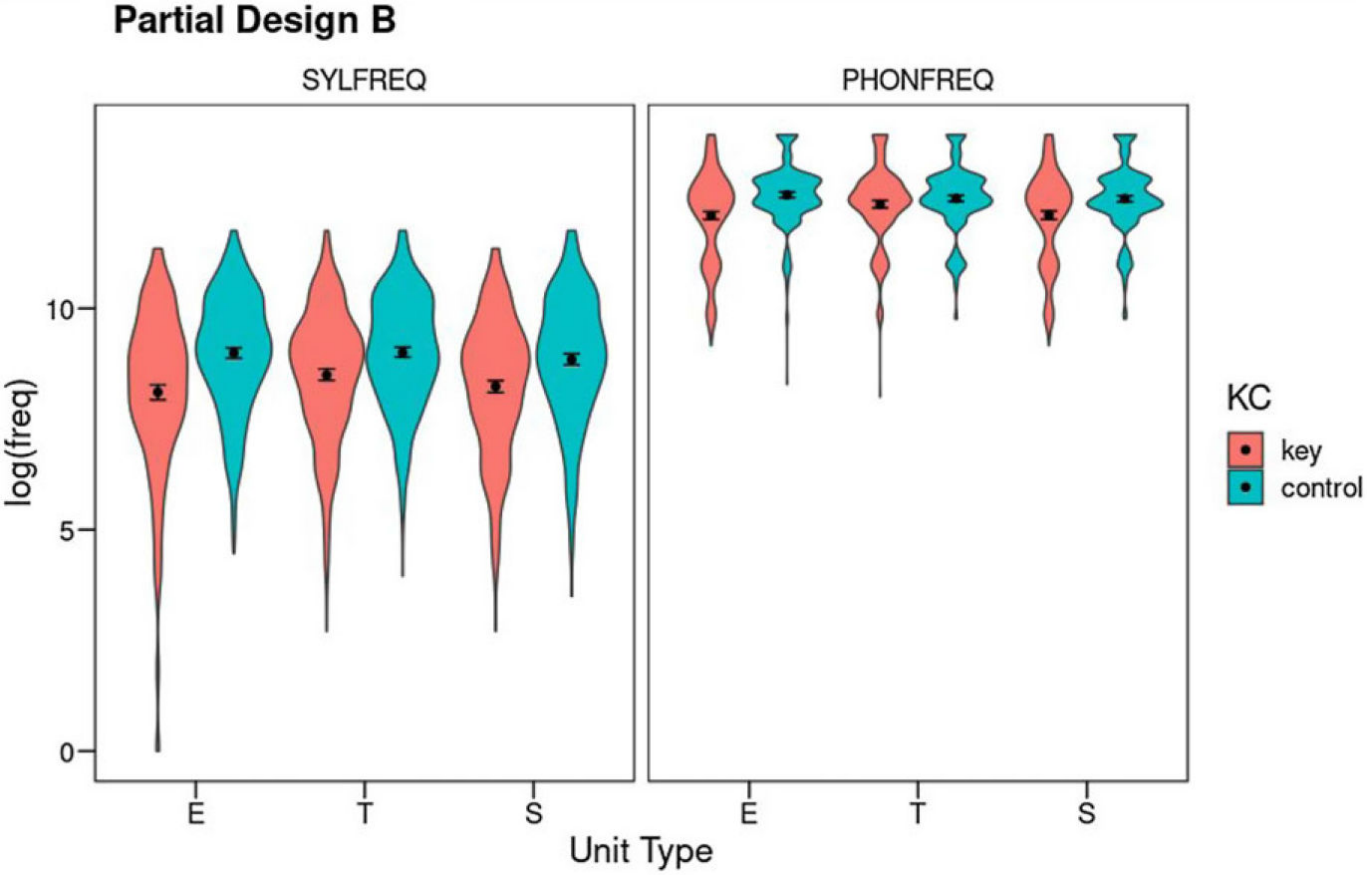
Partial Design B: Mean log_10_ frequency for the factors Unit Type (E: error; T: target; S: source), Key-Control, and Level (SYLFREQ: syllable frequency, PHONFREQ: phoneme frequency). Error bars show 95% confidence intervals using nonparametric bootstrap. The violins show the density of the distributions.

Partial Design B explores only the syllable and phoneme levels, including all three Unit Types (error, target, and source) and the Key-Control contrast. As before, Partial Design B includes errors resulting in nonwords. Total *N* is 502 cases. Figure 3 shows the results (see also Table S11 for means and SDs).

##### Phoneme frequency:

Phoneme frequencies were analyzed as a function of Unit Type and Key-Control, controlling for degree of phoneme underspecification and syllable frequency (see Table S12 for the full output). Note that word frequency cannot be included as a covariate in this design because error nonwords do not have lexical frequency. This is probably of little importance at the phoneme level as prior designs showed that word frequency has a small impact on phoneme frequency. However, it will definitely make the interpretation of the analysis at syllable level more difficult. Both phoneme underspecification (*F*(1,2698.3) = 74.37, *p* < .001, *η*^2^_*p*_ = .03) and syllable frequency (*F*(1,2994.2) = 251.45, *p* < .001, *η*^2^_*p*_ = .08) accounted for significant portions of variance in phoneme frequency. This measure also showed main effects of both factors of interest (Key-Control: *F*(1,2551.8) = 75.22, *p* < .001, *η*^2^_*p*_ = .03; Unit Type: *F*(2,2503.5) = 4.72, *p* = .009, *η*^2^_*p*_ < .01) and their interaction (*F*(2,2502.8) = 12.06, *p* < .001, *η*^2^_*p*_ = .01). Post-hoc Tukey tests showed that both the error and source phonemes were of lower frequency than their controls (error: *t*(1,2534) = −7.57, *p*<.001; source: *t*(1,2518) = −6.55, *p* < .001), but target phonemes were of chance frequency (*t*(1,2514) = −1.22, *p* = .83). Both error phonemes and source phonemes were of lower frequency than targets (error: *t*(1,2509) = −4.52, *p* < .001; source: *t*(1,2506) = 4.85, *p* < .001) whereas there were no differences between the three matched control unit types. Thus, the David effect was substantiated in this design. Because the source phonemes arise at another location as the error, it is not surprising that error phonemes were also less frequent than expected by chance and less frequent than the targets.

##### Syllable frequency:

Syllable frequencies were analyzed as a function of Unit Type and Key-Control, controlling for phoneme frequency (see Table S13). The covariate had a significant effect (*F*(1,3004.4) = 254.00, *p* < .001, *η*^2^_*p*_ = .08). Syllable frequencies showed a Weak Units effect: key units were of lower frequency than their controls (*F*(1,2541.6) = 134.90, *p* < .001, *η*^2^_*p*_ = .05). There was also an effect of Unit Type (*F*(2,2503.8) = 5.59, *p* = .004, *η*^2^_*p*_ < .01). The interaction was marginally significant (*F*(2,2505.7) = 2.92, *p* = .05, *η*^2^_*p*_ < .01). Post-hoc Tukey tests showed that all three unit types were of lower frequency than their corresponding controls (error: *t*(1,2533) = −8.70, *p* < .001; target: *t*(1,2506) = −6.06, *p* < .001; source: *t*(1,2522) = −5.66, *p* < .001). Error syllables had lower frequency than target syllables (*t*(1,2513) = −3.50, *p* = .006), and target and source syllables did not differ in frequency (*t*(1,2512) = 1.79, *p* = .48). Control syllable units did not differ in frequency.

After discounting cross-correlations with measures at the levels of syllable and phoneme (but not at word level), Partial Design B supported prior results from Partial Design A. It showed a clear across-the-board Weak Units effect with the single exception of phoneme targets, as well as a clear David effect at the phoneme level (phoneme sources were of lower frequency than phoneme targets). There was no David effect at the syllable level, which suggests that this effect originates at the phoneme level. Partial Design B also found that error phonemes were of lower frequency than target phonemes, which probably derives from the David effect, because error phonemes are identical to source phonemes and do not change syllable position (so they have similar frequency characteristics). At the syllable level, the finding that error syllables were of lower frequency than target syllables is possibly a result of the uncontrolled influence of word frequency, as the Overall Design showed that error words tend to be of lower frequency than target and source words, and the present Partial Design B was unable to correct for the influence of word frequency on syllable frequency.

## Discussion

Current theories of phonological encoding assume that errors arise because of flawed processing of target units, and therefore, it is a common expectation that target units should be weaker (of lower frequency) than a baseline control. Many models also take the view that source units compete directly with targets in the generation of the error and seem to presuppose that the faulty processing of a target partly originates in stronger processing of the source. Source units should therefore be of higher frequency than targets and probably of higher frequency than a baseline control. Finally, other models suggest that the target competes with the error unit and, therefore, error units should be stronger (of higher frequency) than targets, and perhaps even of higher frequency than a baseline control. These predictions are the same whether we focus on word, syllable, or phoneme units.

The GRLDB allowed us to generate baseline controls, establishing chance levels for word, syllable, and phoneme frequencies. There are a number of different ways that syllable and phoneme frequency can be measured, and we chose one measure for each: the measure that accounted for the most variance, a common practice in statistical analysis. However, the alternative measures would have led to similar results (the supplementary script includes commands for several alternative analyses). The fact that we used token frequency rather than type frequency is not meant to say that type frequency is unimportant; research in phonological development has led to the proposal that phoneme type frequency is the more predictive measure for the order of mastery of phonemes (e.g., Edwards et al., 2015). Type and token frequencies can be differentiated primarily by focusing on subsets of the system where the correlations between the measures break down (e.g., word-initial /ð/ in English, which is low in type frequency but high in token frequency); here, we focus on the entire system.

Because we wanted to examine all error, target, and source frequencies, we ran into a difficulty with errors at the word level: most phonological errors resulted in nonwords, which do not have lexical frequencies. Thus, we ran three models, one that focused just on errors that had real-word outcomes and therefore measurable error-word frequency (the Overall Design), one that included all relevant slips but did not include the frequencies of error units (Partial Design A), and one that included all relevant slips but did not include frequencies at the word level (Partial Design B). Where the models overlapped, they gave essentially the same results, but each added unique information.

Focusing on the findings that were fully convincing statistically, the results reveal a clear and consistent pattern: (1) At the phoneme level, the frequency of target phonemes was indistinguishable from the frequency expected by chance. However, source phonemes were of lower frequency than chance (a Weak Source effect) and of lower frequency than targets (the David effect): weaker sources outcompete stronger targets. Because source phonemes are duplicated in the error, error phonemes were also of lower frequency than chance and of lower frequency than targets. (2) At the syllable level, all three relevant units (error, target, and source) were of lower frequency than chance (a Weak Units effect) without the presence of a David effect (target and source syllables were of comparable frequency). (3) At the word level, there was also an across-the-board Weak Units effect (error, target, and source words of lower-than-chance frequency) with no David effect (target and source words were of comparable frequency). In the Overall Design, restricted to real-word outcomes, error words were of lower frequency than target and source words, and of far lower frequency than baseline control words.

Most models have focused on frequency effects of targets and predict more phonological errors for weak targets, at all levels. Our results for the word level match those predictions: phonological errors are more likely on words of below-average frequency. Insofar as models predict that syllable frequency would have an effect, it would be that low frequency of target syllables would lead to more error, and again, we observed this. However, the models also predict that the target phonemes should be of low frequency. We found that target phonemes do not differ in frequency from their baseline controls. This suggests that frequency effects at the phoneme level have an added level of complexity compared to word and syllable frequency.

Our results about source frequency are clear: phonological errors are more likely with low-frequency source words, low-frequency source syllables, and low-frequency source phonemes, an across-the-board Weak Source effect. A model such as Dell (1986) does not predict this: strongly activated elements in nearby words should be more likely to force their way in to replace targets in other words. We do not know of any processing model that predicts that weak sources are more likely to get involved in errors (though the HiTCH model of Harmon & Kapatsinski, 2021 predicts weak sources in certain circumstances, to which we return in a moment), but there are proposals in which this makes sense. Crystal (1987) did not overtly address this issue but proposed that processing makes use of limited resources that are needed for all levels of processing from lexical access to phonetic encoding, and that errors arise when there is not enough capacity to support what the speaker is trying to produce. Anything that stresses this general resource capacity (such as a low-frequency element) can lead to errors not just on the unit that is causing the problem but on other units as well: when general resources run out, the element that is consuming the most resources is not necessarily the one that is produced incorrectly. A resource-based approach predicts that a weak unit might indirectly cause errors elsewhere in the utterance, but Crystal’s model has not been elaborated enough to be sure of the details, and current models do not incorporate resource limitation effects.

What is needed is an approach that assumes that higher-frequency words (and syllables, if granted psychological reality) are better at both accessing their constituent phonemes (the focus of most processing models) *and* ensuring that those elements appear in the intended locations. Weak sources will thus be more likely to lose control of their constituent phonemes, which then show up in nearby words as phonological errors. One linguistic approach provides a description along these lines, in Bernhardt and Stemberger’s (1998) interpretation of Optimality Theory (Prince & Smolensky, 2004). Prince and Smolensky proposed two constraints on output that they called Integrity (that the phonological elements making up a morpheme must hold together as a chunk) and Contiguity (that those elements must stay in their lexically specified order). If these constraints are violated, a correct element will show up in the wrong location, either within the same word (violating just Contiguity) or in a different word (violating both Contiguity and Integrity). Together, these two constraints lead to the phonemes in a single-morpheme word being output in a single wordform in the lexically specified order. Both constraints ensure that the output is faithful to the lexical representation. Bernhardt and Stemberger (1998) proposed that faithfulness constraints are ranked partly as a function of activation levels, so that they are ranked higher in strong units (which are thus less subject to error) than in weak units (which are thus more subject to error). Among other things, this proposal leads to the result that higher-frequency target words should have more faithful output (with fewer errors) as compared to lower-frequency target words. However, Bernhardt and Stemberger did not anticipate all the consequences of their proposal: if Contiguity and Integrity are violated when one of the word’s constituent phonemes is output in a different word, then a higher-frequency word is more likely to prevent one of its phonemes from straying into a nearby word, as compared to a lower-frequency word. A between-word contextual phonological error always violates both Contiguity and Integrity for the source word. If faithfulness constraints are ranked higher in higher-frequency words than in lower-frequency words, then both targets and sources are predicted to be skewed statistically toward being weak units, as observed in our data. If there are similar constraints for syllables, both the target and source syllables should be of lower frequency than chance. In retrospect, since we expect that strong units will be processed more efficiently and accurately, why would we think that strong units would be less capable of keeping control of the location of their phonological parts? The idea that weak units lose control of their elements, leading to errors in nearby words, is conceptually reasonable. Harmon and Kapatsinski (2021) have recently developed a model in which this is partly the case. Following Elman (1990) and Dell et al. (1993), HiTCH is a recurrent model of production which distinguishes between top-down effects (such as meanings or words) and context effects (what precedes a particular element in the word or sentence). They show how error-driven learning can shift effects of processing from top-down to context units, to a greater and greater degree as production gets further and further into the sentence or word, and the amount of preceding context increases. They show that there is an interaction with frequency, such that high-frequency elements wind up acting more as integrated units and their parts become less likely to act independently, as compared to low-frequency elements. The phonemes of a high-frequency word should be less able to act independently and wind up as an error in a nearby word, so sources should be skewed toward low-frequency words. However, this is not a uniform effect; when context is weakest (such as at the beginning or a sentence, phrase, or word), the top-down effects are strongest and so high-frequency words are more likely than low-frequency words to cause interference elsewhere in the sentence. Our analysis was not designed to test for an interaction. We note that in Spanish, the majority of phonological errors (1ike the majority of consonants and vowels) occur internal to the word rather than at the beginning (with only one phoneme being word-initial), so there may be enough within-word context that HiTCH might predict that source words would tend to be of low frequency. Because HiTCH does not contain syllable units, it would not predict effects of syllable frequency, but would if syllable information could be added to the model. However, research has shown that phonological errors in English tend to be word-initial, which suggests that sources should not be of low frequency, but perhaps should be of high frequency, an interesting prediction for future research. But HiTCH may also predict an interaction between frequency and source elements at the sentence level, so that high-frequency sources are more likely at the beginning of the sentence, but low-frequency sources are more likely later in the sentence (but interacting with the degree of limitation that the context imposes, where, e.g., a verb such as “eat” imposes far more contextual restrictions than a verb such as “see”). The intersentential context may also play a role. Our data were not set up to fully evaluate this sort of interaction between unit frequency and context. Harmon and Kapatsinski’s (2021) model does demonstrate how low-frequency elements can be more likely sources in errors than high-frequency elements, and future research needs to test the predicted interaction between frequency and context.

We also have been able to examine the separate effects of error frequency, but here our results are much more difficult to understand. In the Overall Design (Figure 1), we found that error words are not only lower in frequency than matched controls but also lower in frequency than target and source words. We do not know of any model that would predict this as a basic part of phonological planning. Garrett (1975) maintained that real-word outcomes for phonological errors should arise purely by chance, clearly predicting that the frequency of such wordforms should be at chance. Dell and Reich (1981) reported a lexical bias for their corpus of English errors, but the word frequency of real-word error outcomes was not significantly different from chance. Research on spontaneous error data in Spanish has failed to find a significant lexical bias (del Viso et al., 1991; Pérez et al, 2007), though del Viso et al. reported a slight nonsignificant difference for error words to be of lower frequency than target words. The present study is the first to find that error-word frequency is significantly less than matched controls as well as target and source word frequencies. But if there is no bias toward lexical outcomes, why should the frequency of the lexical outcomes that do occur matter?

We suggest that the below-chance frequency of real-word error outcomes may derive from statistical skewing in the lexicon. Landauer and Streeter (1973) showed that there is a relationship between word frequency and neighborhood density: low-frequency words have on average fewer neighbors, and those neighbors are of lower-than-average frequency. High-frequency words can occur in high-density neighborhoods, because they are relatively easy to perceive correctly, and competition from similar words does not interfere too much with processing. Because low- frequency words are more difficult to perceive correctly, processing suffers from strong negative effects in high-density neighborhoods, from having too many similar competitors, especially if some of the neighbors are of high frequency. The result is that high-frequency words have more neighbors, which can be of any frequency, while low-frequency words have fewer neighbors, which tend to also be of low frequency. In the phonological errors that we examined here, in which target words become error words through the erroneous substitution of one phoneme, the error word is always in the target word’s phonological neighborhood. Because target words are skewed toward being of low frequency, their neighborhoods are skewed toward containing words that are of low frequency, and so real-word error outcomes should have lower average frequency than the lexicon as a whole, as observed. But this by itself does not explain why real-word error outcomes had lower frequencies than target and source words had, and we must leave the issue for resolution in the future.

We found effects of syllable frequency for target, source, and error syllables. The presence of frequency effects for a unit has often been used to argue that that unit is stored, which provides a locus for keeping track of the frequency; this has played a role in the claim that high-frequency word strings are stored (e.g., Tremblay & Baayen, 2010). We believe that the simplest account compatible with the present results is that syllables are stored units, at least in a language of relatively simple syllabic structure and a limited syllabary such as Spanish, though the results may possibly also be compatible with alternative interpretations.

All in all, we have confirmed the expectation that phonological errors occur more on low-frequency target words and have extended this to low-frequency target syllables but have shown that there is no (simple) skewing toward low-frequency phonemes. We have shown that low-frequency source phonemes out-compete target phonemes of higher (chance) frequency (the David effect). We have also shown that source phonemes appear in lower-than-average-frequency syllables and words. Moreover, the resulting error syllables are also of low frequency and, when errors result in real words, those words have even lower lexical frequencies than the target and source words. We believe that the particularly low frequency of lexical results is an artifact of the structure of the lexicon, but the mechanisms remain to be fully spelled out. The challenge now is how to incorporate all these new findings, many of them unexpected, into integrated theories of processing in language production.

This study is of course based on a single set of data, which has been limited to errors that meet a certain set of criteria that allow for relatively straightforward analysis and comparison across all errors in the data. We have shown effects that hold within that subset of the data, but have not, of course, shown that the same effects hold on other subsets of the data; there is always the possibility that the story is far more complex than what we have shown here. Replication using other sets of data is another step, as always.

## Figures and Tables

**Table 1.** Frequency indexes for the syllable /ga/ in the bisyllabic word *gato* (/'.ga.to./), “cat”

Count	Frequency measure
776	Tokens (total number of occurrences) as stressed first syllable in bisyllabic words
35	Types (total number of occurrences in different words) in bisyllabic words as stressed first syllable
776	Tokens as stressed first syllable
35	Types as stressed first syllable
956	Tokens as first syllable in bisyllabic words
48	Types as first syllable in bisyllabic words
2134	Tokens as first syllable
290	Types as first syllable
5382	Tokens as stressed syllable
796	Types as stressed syllable
12,827	Absolute tokens
2012	Absolute types

**Table 2.** Frequency indexes for the phoneme /g/ in the word *gato*

Count	Frequency measure
76,349	Tokens in first onset position
7910	Types (occurrences in different syllables) in first onset position
79,356	Absolute tokens
8387	Absolute types

**Table 3.** Underspecified consonant feature matrix for adult Spanish (i.e., with no default or redundant features listed)

	*Phoneme*																	
*Feature*	p	t	k	b	d	g	č	ǰ	f	θ	s	x	m	n	ɲ	l	r	ɾ
Sonorant													+	+	+	+	+	+
Consonantal																		
Continuant									+	+	+	+					+	+
Nasal													+	+	+			
Lateral																+		
Tense																		+
Laryngeal	✓	✓	✓	✓	✓	✓	✓	✓	✓	✓	✓	✓	✓	✓	✓	✓	✓	✓
Voiced				+	+	+		+										
Spread-glottis																		
Place	✓	✓	✓	✓	✓	✓	✓	✓	✓	✓	✓	✓	✓	✓	✓	✓	✓	✓
Labial	✓			✓					✓				✓					
Round																		
Labiodental																		
Coronal							✓	✓			✓				✓			
Anterior							−	−							−			
Grooved											+							
Dorsal			✓			✓						✓						
Back																		
High																		
Low																		

**Table 4.** Underspecified vowel feature matrix for adult Spanish (i.e., with no default or redundant features listed)

	*Phoneme*				
*Feature*	i	e	u	o	a
Sonorant					
Consonantal	−	−	−	−	−
Continuant					
Nasal					
Lateral					
Tense					
Laryngeal	✓	✓	✓	✓	✓
Voiced					
Spread-glottis					
Place	✓	✓	✓	✓	✓
Labial					
Round					
Labiodental					
Coronal					
Anterior					
Distributed					
Grooved					
Dorsal	✓		✓	✓	✓
Back			+	+	+
High	+		+		
Low					+

**Table 5.** Absolute token frequency and number of cases in which the phoneme acted as source and as target in the sample of speech errors used for present analyses

*Phoneme*	Token frequency	As source	As target
p	236323	22	31
t	391099	44	45
k	364377	36	40
b	226956	18	18
d	451701	20	27
g	79356	12	19
č	19440	35	13
ǰ	49604	20	12
f	61350	16	5
θ	158367	27	21
s	709793	23	49
x	56969	21	16
m	265974	19	17
n	631748	29	28
ɲ	17308	4	1
l	457504	41	42
r	506370	29	39
ɾ	59104	19	12
i	667712	18	15
e	1217917	6	18
u	288240	12	4
o	857490	17	11
a	1165328	14	19

**Table 6.** Sample record from the data file for the slip *Siempre está ab**u**erta la p**u**erta*

Condition	Word	Syllable	Phoneme
Error	abuerta	/buer/	/u/
Control error	confirma	/glen/	/e/
Target	abierta	/bier/	/i/
Control target	hacerlo	/Θien/	/a/
Source	puerta	/puer/	/u/
Control source	poco	/kuan/	/o/

## Footnotes

1 Harmon and Kapatsinski (2021) have recently presented a model that predicts an interaction between frequency and location in the word, such that word-initial phonological error elements should tend to come from high-frequency source words, while error elements later in the word should tend to come from low-frequency source words. We address this below in the discussion.

## References

[R1] Alameda R, & Cuetos F (1995). Diccionario de frecuencias de las unidades lingüistícas del Castellano. Universidad de Oviedo.

[R2] Álvarez CJ, Carreiras M, & Perea M (2004). Are syllables phonological units in visual word recognition? Language and Cognitive Processes, 19(3), 427–452. 10.1080/01690960344000242

[R3] Bates D, Mächler M, Bolker B, & Walker S (2015). Fitting linear mixed-effects models using lme4. Journal of Statistical Software, 67(1), 1–48. 10.18637/jss.v067.i01

[R4] Bernhardt BH, & Stemberger JP (1998). Handbook of phonological development from the perspective of constraint-based nonlinear phonology. Academic Press.

[R5] Ben-Shachar MS, Makowski D, & Lüdecke D (2020). Compute and interpret indices of effect size. CRAN. https://github.com/easystats/effectsize

[R6] Brysbaert M, & Diependaele K (2013). Dealing with zero word frequencies: A review of the existing rules of thumb and a suggestion for an evidence-based choice. Behavior Research Methods, 45(2), 422–430. 10.3758/s13428-012-0270-5 23055175

[R7] Brysbaert M, Mandera P, & Keuleers E (2018). The word frequency effect in word processing: An updated review. Current Directions in Psychological Science, 27(1), 45–50. 10.1177/0963721417727521

[R8] Bybee J. (2006) Frequency of use and the organization of language. Oxford University Press.

[R9] Carreiras M, & Perea M (2004) Naming pseudowords in Spanish: Effects of syllable frequency. Brain and Language, 90, 393–400. 10.1016/j.bandl.2003.12.003 15172555

[R10] Chen J-Y, Lin W-C, & Ferrand L (2003). Masked priming of the syllable in Mandarin Chinese speech production. Chinese Journal of Psychology, 45, 107–120.

[R11] Crystal D. (1987). Towards a ‘bucket’ theory of language disability: Taking account of interaction between linguistic levels. Clinical Linguistics and Phonetics, 1, 7–22.

[R12] del Viso S. (1992). Errores espontáneos del habla y producción del lenguaje. Editorial de la Universidad Complutense de Madrid.

[R13] del Viso S, Igoa JM, & García-Albea JE (1991). On the autonomy of phonological encoding: Evidence from slips of the tongue in Spanish. Journal of Psycholinguists Research, 20, 161–185.

[R14] Dell GS (1986). A spreading-activation theory of retrieval in sentence production. Psychological Review, 93, 283–321. 3749399

[R15] Dell GS (1990). Effects of frequency and vocabulary type on phonological speech errors. Language and Cognitive Processes, 5, 313–349.

[R16] Dell GS, Juliano C, & Govindjee A (1993). Structure and content in language production: A theory of frame constraints in phonological speech errors. Cognitive Science, 17, 149–195.

[R17] Dell GS, & Reich PA (1981). Stages in sentence production: An analysis of speech error data. Journal of Verbal Learning and Verbal Behavior, 20, 611–629.

[R18] Edwards J, Beckman ME, & Munson B (2015). Frequency effects in phonological acquisition. Journal of Child Language, 42(2), 306–311. 25644416 10.1017/S0305000914000634 PMC4318350

[R19] Elman JL (1990). Finding structure in time. Cognitive Science, 14, 179–211.

[R20] Francis WN, & Kučera H (1982). Frequency analysis of English usage: Lexicon and grammar. Houghton Mifflin.

[R21] Fromkin VA (1971). The Non-Anomalous Nature of Anomalous Utterances. Language, 47(1), 27–52. 10.2307/412187

[R22] Garrett M (1975). The analysis of sentence production. In Bower G (Ed.), Psychology of learning and motivation (Vol. 9, pp. 133–177). Academic Press.

[R23] Harmon Z, & Kapatsinski V (2021). A theory of repetition and retrieval in language production. Psychological Review, 128(6), 1112–1144. 34242049 10.1037/rev0000305

[R24] Kuznetsova A, Brockhoff PB, & Christensen RHB (2017). lmerTest Package: Tests in linear mixed effects models. Journal of Statistical Software, 82(13). 10.18637/jss.v082.i13

[R25] Landauer TK, & Streeter LA (1973). Structural differences between common and rare words: Failure of equivalence assumptions for theories of word recognition. Journal of Verbal Learning & Verbal Behavior, 12(2), 119–131. 10.1016/S0022-5371(73)80001-5

[R26] Laganaro M, & Alario F-X (2006) On the locus of the syllable frequency effect in speech production. Journal of Memory and Language, 55, 178–196.

[R27] Lenths R. (2019). emmeans: Estimated Marginal Means, aka Feast-Squares Means. CRAN. https://CRAN.R-project.org/package=emmeans

[R28] Levelt WJM, & Wheeldon LR (1994). Do speakers have access to a mental syllabary? Cognition, 50, 239–269. 8039363 10.1016/0010-0277(94)90030-2

[R29] Levitt AG, & Healy AF (1985). The roles of phoneme frequency, similarity, and availability in the experimental elicitation of speech errors. Journal of Memory and Language, 24, 717–733.

[R30] MacKay DG (1970). Spoonerisms: The structure of errors in the serial order of speech. Neuropsychologia, 8, 323–350. 5522566 10.1016/0028-3932(70)90078-3

[R31] Motley MT, Baars BJ, & Camden CT (1983). Experimental verbal slips studies: A review and an editing model of language encoding. Communication Monographs, 50, 79–101.

[R32] Nickels LA, & Howard D (2004). Dissociating effects of number of phonemes, number of syllables, and syllabic complexity on word production in aphasia: It’s the number of phonemes that counts. Cognitive Neuropsychology, 21, 57–78. 21038191 10.1080/02643290342000122

[R33] Nooteboom SG (1969). The tongue slips into patterns. In Sciarone AG (Ed.), NOMEN: Leyden studies in linguistics and phonetics. Mouton (pp. 114–132).

[R34] Pérez E, Santiago J, Palma A, & O’Seaghdha PG (2007). Perceptual bias in speech error data collection: Insights from Spanish speech errors. Journal of Psycholinguists Research, 36, 207–235. 10.1007/s10936-006-9042-7 17186384

[R35] Prince A, & Smolensky P (2004). Optimality theory: Constraint interaction in generative grammar. Blackwell.

[R36] R Core Team. (2018). R: A language and environment for statistical computing. R Foundation for Statistical Computing.

[R37] Santiago J, Justicia F, Palma A, Huertas D, & Gutiérrez-Palma N (1996). LEX I and II: Two data-bases of surface word forms for psycholinguistic research in Spanish. Behavior Research Methods, Instruments, & Computers, 28(3), 418–426. 10.3758/BF03200522

[R38] Santiago J, Pérez E, Palma A, & Stemberger JP (2007). Syllable, word, and phoneme frequency effects in Spanish phonological speech errors: The David effect on the source of the error. In Carson TS and Ferreira V (Eds.), The state of the art in speech error research: Proceedings of the LSA Institute Workshop. MIT working papers in linguistics, vol. 53 (pp. 265–303).

[R39] Schiller NO (1998). The effect of visually masked syllable primes on the naming latencies of words and pictures. Journal of Memory and Language, 39, 484–507.

[R40] Shattuck-Hufnagel S (1979). Speech error evidence for a serial ordering mechanism in sentence production. In Cooper WE and Walker ECT (Eds.), Sentence processing: Psycholinguists studies presented to Merrill Garrett. Erlbaum (pp. 295–342).

[R41] Shattuck-Hufnagel S, & Klatt DH (1979). The limited use of distinctive features and markedness in speech production: Evidence from speech error data. Journal of Memory and Language, 18, 41–55.

[R42] Stemberger JP (1991a). Apparent anti-frequency effects in language production: The addition bias and phonological underspecification. Journal of Memory and Language, 30, 161–185.

[R43] Stemberger JP (1991b). Radical underspecification in language production. Phonology, 8, 73–112.

[R44] Stemberger JP (2009). Preventing perseveration in language production. Language and Cognitive Processes, 24(10), 1431–1470.

[R45] Stemberger JP, & MacWhinney B (1986). Frequency and the lexical storage of regularly inflected forms. Memory & Cognition, 14, 17–26. 3713503 10.3758/bf03209225

[R46] Tremblay A, & Baayen H (2010). Holistic processing of regular four-word sequences: A behavioural and ERP study of the effects of structure, frequency, and probability on immediate free recall. In Wood D (Ed.), Perspectives on formulaic language: Acquisition and communication (pp. 151–173). Continuum.

[R47] Umeda N, & Kahn D (1982). Frequency of occurrence of two- and three-word sequences in English. Journal of the Acoustical Society of America, 72, 2031–2033.

[R48] Vitevitch MS, & Luce PA (2015). Phonological neighborhood effects in spoken word perception and production. Annual Review of Linguistics, 2, 75–94.

[R49] Zipf GK (1949). Human behavior and the principle of least effort: An introduction to human ecology. Addison-Wesley Press.

